# *tert*-Butyl imidazole-1-carboxyl­ate

**DOI:** 10.1107/S1600536809003110

**Published:** 2009-01-28

**Authors:** Tobias Kerscher, Tanja Prommnitz, Peter Klüfers, Peter Mayer

**Affiliations:** a Ludwig-Maximilians Universität, Department Chemie und Biochemie, Butenandtstrasse 5–13 (Haus D), 81377 München, Germany

## Abstract

In the title compound, C_8_H_12_N_2_O_2_, mol­ecules are inter­connected by weak C—H⋯O contacts with H⋯O distances of 2.30 Å, resulting in the formation of chains along [100]. According to graph-set analysis, the unitary descriptor of these chains is *C*(5). In addition, there are π–π stacking inter­actions between pyrazole rings (centroid distance = 3.878 Å and ring plane distance = 3.26 Å).

## Related literature

The title compound is a well known organic compound and was prepared according to a recently published procedure (Jia *et al.*, 2007 ▶). For details of graph-set analysis see: Etter *et al.* (1990 ▶); Bernstein *et al.* (1995 ▶).
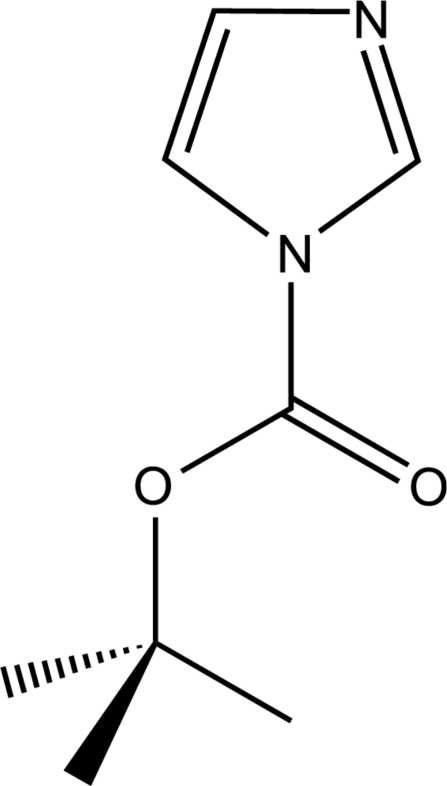


## Experimental

### 

#### Crystal data

C_8_H_12_N_2_O_2_
*M* *_r_* = 168.19
Monoclinic, 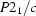

*a* = 5.9952 (2) Å
*b* = 13.2507 (4) Å
*c* = 11.5564 (4) Å
β = 94.201 (2)°
*V* = 915.58 (5) Å^3^
*Z* = 4
Mo *K*α radiation
μ = 0.09 mm^−1^
*T* = 200 (2) K
0.50 × 0.38 × 0.38 mm

#### Data collection

Nonius KappaCCD diffractometer
Absorption correction: none
6875 measured reflections
2097 independent reflections
1650 reflections with *I* > 2σ(*I*)
*R*_int_ = 0.026

#### Refinement

*R*[*F* ^2^ > 2σ(*F* ^2^)] = 0.040
*wR*(*F* ^2^) = 0.104
*S* = 1.07
2097 reflections
113 parameters
H-atom parameters constrained
Δρ_max_ = 0.17 e Å^−3^
Δρ_min_ = −0.16 e Å^−3^

### 

Data collection: *COLLECT* (Nonius, 2004 ▶); cell refinement: *SCALEPACK* (Otwinowski & Minor, 1997 ▶); data reduction: *SCALEPACK* and *DENZO* (Otwinowski & Minor, 1997 ▶); program(s) used to solve structure: *SHELXS97* (Sheldrick, 2008 ▶); program(s) used to refine structure: *SHELXL97* (Sheldrick, 2008 ▶); molecular graphics: *ORTEP-3* (Farrugia, 1997 ▶); software used to prepare material for publication: *SHELXL97*.

## Supplementary Material

Crystal structure: contains datablocks I, global. DOI: 10.1107/S1600536809003110/bt2854sup1.cif

Structure factors: contains datablocks I. DOI: 10.1107/S1600536809003110/bt2854Isup2.hkl

Additional supplementary materials:  crystallographic information; 3D view; checkCIF report

## Figures and Tables

**Table 1:** Hydrogen-bond geometry (Å, °)

*D*—H⋯*A*	*D*—H	H⋯*A*	*D*⋯*A*	*D*—H⋯*A*
C1—H1⋯O2^i^	0.95	2.30	3.1949 (16)	156

Symmetry code: (i) 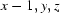.

**Fig. 1.**
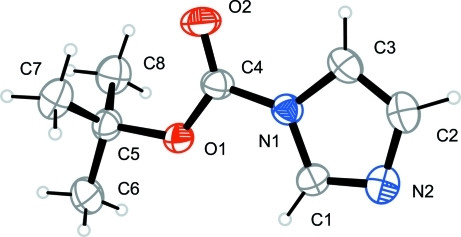
The molecular structure of the title compound with atom labels and anisotropic displacement ellipsoids (drawn at 50% probability level) for non-H atoms.

**Fig. 2.**
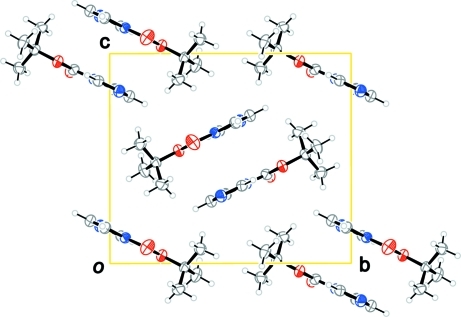
The packing of the title compound, viewed along [100].

**Fig. 3.**
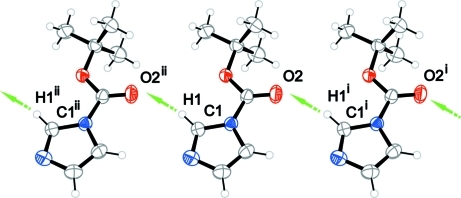
Weak C—H···O interactions lead to chain-like structures in the crystal structure along [100] shown here normal to [001]. Symmetry codes: (i) x + 1, y, z; (ii) x - 1, y, z.

**Fig. 4.**
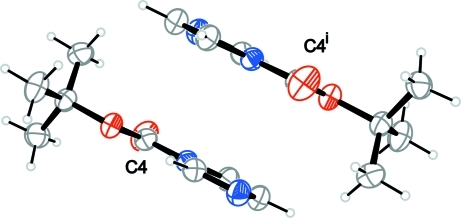
π interaction leads to dimeric units shown here normal to [100]. Symmetry code: (i) -x, -y, -z.

**Fig. 5.**
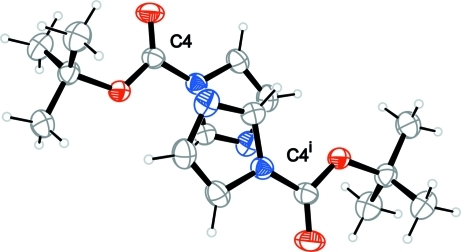
The dimeric units formed by π interaction are shifted, so that only half of the imidazole rings overlap. Two of the imidazole rings are shown here, normal to [001]. Symmetry code: (i) -x, -y, -z.

## supplementary crystallographic information

### Comment

The title compound was synthesized in a multistep sythesis in an attempt to create new complexing ligands.

The molecule is an imidazole protected by the *tert*-butyloxycarbonyle (Boc) group in the 1 position (see Fig. 1).

The crystal packing is shown in Fig. 2. In the crystal, weak C—H···O contacts along [100] which can be described according to graph-set analysis (Etter *et al.*, 1990; Bernstein *et al.*, 1995) with a unitary *C*(5) descriptor (see Fig. 3), lead to chain like structures of dimeric units which are formed by π-type interaction of two imidazole rings (see Fig. 4). The two imidazole rings are separated by about 3.26 Å and shifted, which results in only about half of one ring overlapping with the other ring (see Fig. 5).

Interestingly the imidazoles do not form longer strands of π-type interacting aromatic systems but only dimeric units which might be due to the large space occupied by the Boc protecting group which leads to separate strands of C—H···O bridged dimeric units (see Fig. 2).

### Experimental

Boc_2_O was reacted solvent free with one equivalent of imidazole. After the CO_2_ gas evolution had finished, the byproduct, *t*-butanole, was removed by fine vacuum and big colorless crystals of the title compound were obtained.

### Refinement

H atoms were placed in calculated positions (C—H 0.95 Å for aromatic C atoms and C—H 0.98 Å for methyl C atoms) and were included in the refinement in the riding model approximation with U(H) set to 1.2*U*_eq_(C) for aromatic C atoms and 1.5*U*_eq_(C) for methyl C atoms.

### Crystal data

**Table:**

C_8_H_12_N_2_O_2_	*F*(000) = 360
*M**_r_* = 168.19	*D*_x_ = 1.220 Mg m^−^^3^
Monoclinic, *P*2_1_/*c*	Mo *K*α radiation, λ = 0.71073 Å
Hall symbol: -P 2ybc	Cell parameters from 3660 reflections
*a* = 5.9952 (2) Å	θ = 3.1–27.5°
*b* = 13.2507 (4) Å	µ = 0.09 mm^−^^1^
*c* = 11.5564 (4) Å	*T* = 200 K
β = 94.201 (2)°	Block, colourless
*V* = 915.58 (5) Å^3^	0.50 × 0.38 × 0.38 mm
*Z* = 4	

### Data collection

**Table:**

Nonius KappaCCD diffractometer	1650 reflections with *I* > 2σ(*I*)
Radiation source: rotating anode	*R*_int_ = 0.026
MONTEL, graded multilayered X-ray optics	θ_max_ = 27.5°, θ_min_ = 3.4°
CCD; rotation images; thick slices scans	*h* = −7→7
6875 measured reflections	*k* = −17→17
2097 independent reflections	*l* = −14→15

### Refinement

**Table:**

Refinement on *F*^2^	Secondary atom site location: difference Fourier map
Least-squares matrix: full	Hydrogen site location: inferred from neighbouring sites
*R*[*F*^2^ > 2σ(*F*^2^)] = 0.040	H-atom parameters constrained
*wR*(*F*^2^) = 0.104	*w* = 1/[σ^2^(*F*_o_^2^) + (0.0396*P*)^2^ + 0.2307*P*] where *P* = (*F*_o_^2^ + 2*F*_c_^2^)/3
*S* = 1.07	(Δ/σ)_max_ < 0.001
2097 reflections	Δρ_max_ = 0.17 e Å^−^^3^
113 parameters	Δρ_min_ = −0.15 e Å^−^^3^
0 restraints	Extinction correction: *SHELXL97* (Sheldrick, 2008)
Primary atom site location: structure-invariant direct methods	Extinction coefficient: 0.063 (6)

### Special details

**Table:**

Refinement. Hydrogen atoms were placed in calculated positions (C–H 0.95 Å for aromatic C atoms and C–H 0.98 Å for methyl C atoms) and were included in the refinement in the riding model approximation with U(H) set to 1.2 *U*_eq_(C) for aromatic C atoms and 1.5 *U*_eq_(C) for methyl C atoms.

### Fractional atomic coordinates and isotropic or equivalent isotropic displacement parameters (Å^2^)

**Table:**

	*x*	*y*	*z*	*U*_iso_*/*U*_eq_	
O1	0.15042 (14)	0.21450 (6)	0.03780 (8)	0.0356 (3)	
O2	0.50241 (15)	0.15584 (8)	0.07833 (10)	0.0527 (3)	
N1	0.19880 (17)	0.06402 (8)	0.12264 (9)	0.0329 (3)	
N2	−0.0623 (2)	−0.03954 (9)	0.17875 (11)	0.0442 (3)	
C1	−0.0253 (2)	0.04699 (10)	0.13009 (11)	0.0359 (3)	
H1	−0.1397	0.0927	0.1031	0.043*	
C2	0.1485 (2)	−0.08085 (11)	0.20408 (13)	0.0450 (4)	
H2	0.1750	−0.1445	0.2404	0.054*	
C3	0.3103 (2)	−0.01943 (10)	0.17071 (12)	0.0410 (3)	
H3	0.4671	−0.0307	0.1784	0.049*	
C4	0.3034 (2)	0.14897 (10)	0.07701 (11)	0.0350 (3)	
C5	0.2187 (2)	0.30947 (10)	−0.01876 (12)	0.0373 (3)	
C6	−0.0052 (3)	0.35703 (12)	−0.05516 (15)	0.0526 (4)	
H6A	−0.0861	0.3708	0.0138	0.079*	
H6B	−0.0929	0.3107	−0.1065	0.079*	
H6C	0.0187	0.4204	−0.0963	0.079*	
C7	0.3515 (3)	0.37416 (11)	0.06973 (13)	0.0485 (4)	
H7A	0.4941	0.3410	0.0926	0.073*	
H7B	0.2661	0.3830	0.1382	0.073*	
H7C	0.3801	0.4402	0.0357	0.073*	
C8	0.3472 (3)	0.28433 (12)	−0.12317 (13)	0.0501 (4)	
H8A	0.2566	0.2400	−0.1756	0.075*	
H8B	0.4871	0.2501	−0.0974	0.075*	
H8C	0.3812	0.3467	−0.1640	0.075*	

### Atomic displacement parameters (Å^2^)

**Table:**

	*U*^11^	*U*^22^	*U*^33^	*U*^12^	*U*^13^	*U*^23^
O1	0.0293 (5)	0.0336 (5)	0.0438 (5)	−0.0001 (3)	0.0018 (4)	0.0068 (4)
O2	0.0279 (5)	0.0595 (7)	0.0709 (7)	0.0038 (4)	0.0052 (5)	0.0188 (6)
N1	0.0322 (6)	0.0335 (6)	0.0329 (5)	0.0020 (4)	0.0007 (4)	0.0020 (4)
N2	0.0469 (7)	0.0399 (6)	0.0458 (7)	−0.0045 (5)	0.0035 (5)	0.0022 (5)
C1	0.0334 (7)	0.0376 (7)	0.0366 (7)	−0.0013 (5)	0.0017 (5)	−0.0004 (5)
C2	0.0549 (9)	0.0359 (7)	0.0436 (8)	0.0016 (6)	0.0004 (6)	0.0049 (6)
C3	0.0424 (8)	0.0385 (7)	0.0413 (7)	0.0078 (6)	−0.0022 (6)	0.0036 (6)
C4	0.0312 (7)	0.0384 (7)	0.0353 (6)	0.0023 (5)	0.0016 (5)	0.0028 (5)
C5	0.0389 (7)	0.0338 (7)	0.0392 (7)	−0.0034 (5)	0.0036 (5)	0.0049 (5)
C6	0.0507 (9)	0.0407 (8)	0.0654 (10)	0.0045 (7)	−0.0025 (7)	0.0133 (7)
C7	0.0532 (9)	0.0456 (8)	0.0470 (8)	−0.0127 (7)	0.0054 (7)	−0.0033 (7)
C8	0.0600 (10)	0.0514 (9)	0.0397 (8)	−0.0053 (7)	0.0093 (7)	0.0038 (7)

### Geometric parameters (Å, °)

**Table:**

O1—C4	1.3187 (15)	C5—C6	1.5144 (19)
O1—C5	1.4892 (15)	C5—C7	1.5147 (19)
O2—C4	1.1958 (15)	C5—C8	1.515 (2)
N1—C1	1.3714 (16)	C6—H6A	0.9800
N1—C3	1.3870 (16)	C6—H6B	0.9800
N1—C4	1.4094 (17)	C6—H6C	0.9800
N2—C1	1.3032 (17)	C7—H7A	0.9800
N2—C2	1.3886 (19)	C7—H7B	0.9800
C1—H1	0.9500	C7—H7C	0.9800
C2—C3	1.344 (2)	C8—H8A	0.9800
C2—H2	0.9500	C8—H8B	0.9800
C3—H3	0.9500	C8—H8C	0.9800
			
C4—O1—C5	120.04 (10)	C6—C5—C8	111.24 (12)
C1—N1—C3	106.82 (11)	C7—C5—C8	112.92 (12)
C1—N1—C4	128.28 (10)	C5—C6—H6A	109.5
C3—N1—C4	124.90 (11)	C5—C6—H6B	109.5
C1—N2—C2	104.88 (11)	H6A—C6—H6B	109.5
N2—C1—N1	111.73 (11)	C5—C6—H6C	109.5
N2—C1—H1	124.1	H6A—C6—H6C	109.5
N1—C1—H1	124.1	H6B—C6—H6C	109.5
C3—C2—N2	111.44 (12)	C5—C7—H7A	109.5
C3—C2—H2	124.3	C5—C7—H7B	109.5
N2—C2—H2	124.3	H7A—C7—H7B	109.5
C2—C3—N1	105.13 (12)	C5—C7—H7C	109.5
C2—C3—H3	127.4	H7A—C7—H7C	109.5
N1—C3—H3	127.4	H7B—C7—H7C	109.5
O2—C4—O1	128.48 (12)	C5—C8—H8A	109.5
O2—C4—N1	121.79 (11)	C5—C8—H8B	109.5
O1—C4—N1	109.72 (10)	H8A—C8—H8B	109.5
O1—C5—C6	101.94 (10)	C5—C8—H8C	109.5
O1—C5—C7	109.24 (11)	H8A—C8—H8C	109.5
C6—C5—C7	111.30 (12)	H8B—C8—H8C	109.5
O1—C5—C8	109.63 (11)		
			
C2—N2—C1—N1	−0.07 (15)	C5—O1—C4—N1	−177.82 (10)
C3—N1—C1—N2	0.14 (15)	C1—N1—C4—O2	178.00 (13)
C4—N1—C1—N2	−179.21 (12)	C3—N1—C4—O2	−1.2 (2)
C1—N2—C2—C3	−0.04 (16)	C1—N1—C4—O1	−0.98 (18)
N2—C2—C3—N1	0.12 (16)	C3—N1—C4—O1	179.77 (11)
C1—N1—C3—C2	−0.15 (14)	C4—O1—C5—C6	176.89 (11)
C4—N1—C3—C2	179.23 (12)	C4—O1—C5—C7	−65.27 (15)
C5—O1—C4—O2	3.3 (2)	C4—O1—C5—C8	58.95 (15)

### Hydrogen-bond geometry (Å, °)

**Table:**

*D*—H···*A*	*D*—H	H···*A*	*D*···*A*	*D*—H···*A*
C1—H1···O2^i^	0.95	2.30	3.1949 (16)	156

Symmetry codes: (i) *x*−1, *y*, *z*.
